# Bryan Lask, FRCPsych: Formerly Emeritus Professor of Child and Adolescent Psychiatry, St George's University of London

**DOI:** 10.1192/pb.bp.115.053421

**Published:** 2016-06

**Authors:** Philip Graham

**Figure:**
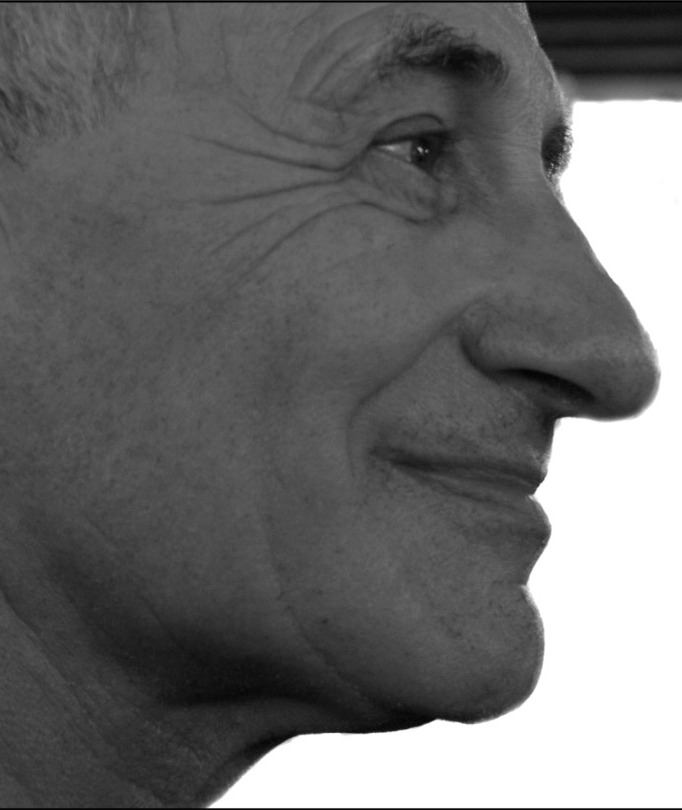


Bryan Lask died aged 74 on 25 October 2015 after a long illness. He continued to work, teach, write and research until 6 months before his death. He was a pioneer child and adolescent psychiatrist with an international reputation for his work on eating disorders, especially those occurring in children and adolescents. After 12 years of investigating and treating the families and psychological states of these patients – usually, but by no means always girls – he turned his attention to the possibility that there might be a brain-based explanation for these conditions. In the mid-1990s, with his colleague Isky Gordon, a leading paediatric radiologist, he embarked on a series of studies of brain functioning. Alongside these, in collaboration with research psychologists, he carried out numerous studies of neuropsychological function. It had previously been assumed that the causes of anorexia nervosa lay entirely in the personalities and upbringing of those with this disorder. Inevitably, parents were made to feel guilty at the thought that they might have been responsible for their children's eating disorders.

Lask and Gordon were able to demonstrate changes in regional blood flow in the brains of girls with eating disorders. With his long-standing colleague, Kenneth Nunn, an Australian child psychiatrist, Lask developed a neurobiological theory focusing on the insula, a deep-lying structure in the brain, as central to the development of these conditions. These studies were brought together in a book entitled *Eating Disorders and the Brain*, edited by Bryan Lask and Ian Frampton. More than anything else, Lask aimed to reduce the stigma and blame associated with eating disorders by highlighting that they were not a choice but an affliction. Following training in London at Great Ormond Street Hospital for Children (formerly the Hospital for Sick Children), Nunn has had a successful academic career in Australia but he and Lask remained close friends and colleagues over the years.

From his earliest days as a consultant child psychiatrist, Lask had shown an unusual ability to bring scientific rigour to his subject while retaining genuine enthusiasm for finding ways of treating illness. At a time when treatment studies in the field were rare, he carried out a controlled investigation of family therapy in patients with childhood asthma, demonstrating benefit to those who received the treatment. He showed an early interest in childhood-onset anorexia nervosa and with his colleague Rachel Bryant-Waugh, a gifted research-oriented clinical psychologist, he carried out an important series of clinical and follow-up studies of this condition. It became apparent from these studies just how crippling early-onset anorexia nervosa could be. An article published in 1987 by the team he led described the first large study of young children with anorexia nervosa. There were 48 children, 13 boys and 35 girls, aged between 7 and 13; half had not reached even the first stages of puberty. They showed the typical symptoms of the condition: food refusal, severe loss of weight, depression, fear of fatness, distorted body image, self-induced vomiting, excessive physical exercise, obsessional behaviour, bingeing and laxative misuse. The existence of the condition in children before puberty and in boys gave the lie to the idea that this was necessarily a disease of adolescence or that it arose from a fear of growing up to be a fully mature woman. Childhood-onset anorexia nervosa was revealed to be a serious condition. A follow-up 7 years later revealed that 10 children had remained moderately or severely impaired in their everyday lives, and 2 had died.

He also had a gift for identifying previously unrecognised disorders. In 1991, with colleagues, he published the first clinical description of so-called pervasive refusal syndrome, a condition in which children between the ages of 9 and 15 refuse to eat, drink, walk or talk for months or even years at a time. One of the four children described was a 9-year-old girl transferred from a paediatric ward. After she had developed normally into an intelligent, hard-working, popular girl, she had become increasingly withdrawn. She eventually stopped speaking, making only high-pitched moans. On admission to the children's psychiatric ward she was alert and showed interest in her surroundings but avoided eye contact by covering her eyes whenever an adult came near her. She refused to walk or care for herself and resisted all physical contact, including any form of feeding. After 18 months of group and individual therapy and encouragement from nursing staff, she was eating normally and was mobile but refused to go home. Reasons for her rejection of home were suspected but nothing could be proved. Eventually, she was placed in a small children's home, while fully active. Pervasive refusal disorder is now identified as a major syndrome, occurring, for example, in refugee children who have given up hope at a time when they feel their parents have also given up hope.

Lask's teaching hospital appointments gave him a role as mentor, teacher, trainer and facilitator which he grasped with enthusiasm. He had an extraordinary gift of being able to encourage, guide and support, and used this to ensure that a whole generation of active, inspired, effective and productive clinicians and researchers was added to the field. Another of Lask's outstanding characteristics was his ability to communicate with children and their families as well as with the wider public. The week after his appearance in October 2013 on the BBC Radio 4 programme, ‘Inside Health: Talking about eating disorders’, the presenter, Mark Porter, reported that his producer had had so much positive feedback from listeners on Lask's contribution that he was thinking of setting up a Bryan Lask fan club!

Bryan Lask was born on 18 February 1941. He was brought up in London, the eldest of the three sons of Rita and Aaron Lask, a general practitioner well known for his interest in psychosomatic medicine. He was educated at St Paul's School and decided on a career in medicine. He had great difficulty in passing the examination in physics necessary for admission to medical school. After two failures, only St Bartholomew's, London, would consider him but they required a 50% pass in physics. He sat the examination and with great anxiety, on 21 December 1960, before hearing the result, he plucked up the courage to ring the Professor of Physics. This happened to be Joseph Rotblat, a nuclear physicist who had worked on but then withdrawn from the Manhattan Project and who later won the Nobel Peace Prize. According to Lask a ‘sympathetic and kindly eastern European accent emerged and said “Lask, yes indeed Lask. I do have your result here. You obtained 49% but as Christmas is approaching we'll call it 50%”.’

After medical qualification in 1966, he trained in psychiatry and then child psychiatry at the Maudsley Hospital and Great Ormond Street Hospital for Children, London. He met his wife, Judith – a social worker who later achieved prominence in her own field – when he was a junior doctor and she a young social worker. They ran a therapeutic group in the Maudsley children's department together. Lask was appointed consultant child psychiatrist in the department of psychological medicine at the hospital in 1975. There he entered fully into all aspects of the work of a very busy department. He carried out research and published on the psychological aspects of cystic fibrosis and heart transplantation in children but, in collaboration with Rachel Bryant-Waugh, his main interest was in the establishment of the first nationally recognised eating disorders clinic for children and adolescents.

In 1998 he moved to St George's Hospital where he was appointed to a Chair in Child and Adolescent Psychiatry. From 2004 to 2011 he worked as a Visiting Professor and Research Director at the Regional Eating Disorders Service, University of Oslo, Norway, where he set up a major research programme. His international standing was confirmed by his election to President of the Eating Disorders Research Society (2010–2011). He received a Lifetime Achievement Award from the Academy for Eating Disorders (2011), as well as an Outstanding Leadership and Service Award from the Eating Disorders Research Society (2014). As recently as 2012 he was appointed the founding editor of a new journal, *Advances in Eating Disorders – Theory, Research and Practice*. He was constantly trying to encourage plain English, develop practical approaches, dismantle professional pretence and avoid medical jargon. In 1985 he published an editorial in the *Journal of Family Therapy* entitled ‘Jargon, ambiguity, pomposity and other pests’, deploring the style of much academic literature in the field.

It is not widely known that from 1977 and for the rest of his life, Lask battled with the complications of treatment for bowel cancer. His condition required frequent admission to hospital, often for days or weeks at a time. He wrote a paper on the psychological effects of stoma surgery, but his own courage and extraordinary energy in the face of long-standing illness were remarkable. He maintained a punishing schedule of teaching and presenting keynote addresses at international conferences. He became separated, but not estranged, from Judith who gave him great love and care throughout his life and his illness. He died after saying goodbye to her and is also survived by his two sons – Gideon, the chief executive officer and founder of multiple technology businesses, and Adam, Corporate Health and Wellbeing Consultant – as well as four greatly loved grandchildren, Raffi, Lucas, Cassius and Lila.

When Bryan Lask received his lifetime achievement award from the Academy for Eating Disorders in 2011, it was said of him that he was ‘a big, warm-hearted, generous character whose contribution to the field represented a truly extraordinary lifetime of commitment and passion for his work’. All his colleagues would endorse that judgement.

